# Introductory Lecture, at the Opening of the Thirteenth Annual Session of Rush Medical College, Nov. 5th, 1855

**Published:** 1855-12

**Authors:** Daniel Brainard

**Affiliations:** Professor of Surgery and President of the College


THE NORTH-WESTERN

MEDICAL AND SURGICAL JOURNAL.

NEW SERIES.

VOL. IV. DECEMBER, 1855. NO. 12.

### ORIGINAL COMMUNICATIONS.

ART. I.—*Introductory Lecture, at the opening of the thirteenth Annual Session of Rush Medical College, Nov. 5th,* 1855.—By DANIEL BRAINARD, M. D. Professor of Surgery and President of the College.

GENTLEMEN OF THE MEDICAL CLASS:

The present occasion seems to constitute an epoch in the history of the Medical College of Chicago, in which, it will I hope, be considered not out of place to pass in review the past history of the Institution, take a survey of its present condition, and cast a glance at its prospects for the future.

It is now eleven years since, on the occasion of the finishing of our first building, I addressed the medical class and the public, at the opening of the second annual course of lectures, and I well remember, thinking myself called upon at that time to enter into an enumeration of the reasons which had induced me in connection with my colleagues and friends, to attempt the building up of a Medical Institution in this city; for sanguine as were the hopes at that time, concerning the prospects of this city, it is not to be denied that many persons here regarded the attempt as premature; and abroad we have reason to know that the undertaking was looked upon as chimerical, likely to end in failure; and if successful, destined to add another to the number of medical schools, already too great, in which neither competent teachers nor suitable means of instruction were to be found. In order to correct and counteract these opinions, I entered on the occasion referred to, into an elaborate enumeration of reasons deemed suitable to justify the undertaking. The growth of the city, and its certain prospect of becoming the metropolis of a large section of country, the fertility of the country itself, and the certainty of its being soon filled with a dense population, the probability of securing the services of competent, scientific men as teachers, and of procuring suitable apparatus, and means of illustrating the lectures on the various branches, together with the certainty that the city would afford, in hospitals to be established, sufficient means of clinical instruction—such were the topics to which I endeavored to direct the attention of the audience.

It is probable that this attempt met with but very partial success, and that many went away as they came, incredulous in regard to the feasibility of an enterprise, of which indeed, time alone could determine the practicability and the wisdom.

The lapse of twelve years, has shown the entire feasibility or the impracticability of many a cherished scheme—no one at this day can pretend to have foreseen what has happened in this city and the surrounding country, yet in regard to this undertaking we may now safely say, that time has demonstrated the soundness of the reasons on which it was based, vindicated the prudence of those who originated it, and shown abundantly, that to hold a position of honor and usefulness, it had need only to keep pace with the growth of our city and of the State.

The first idea of the establishment of a medical college in Chicago, dates as far back as 1836. In the autumn of that year, in connection with the late J. C. Goodhue, of Rockford in this State, then a resident of this city, I drew up the act of incorporation of this college, and had it presented to the Legislature, at its session in Vandalia, the subsequent winter. Nearly all the innumerable applications for charters of every kind were at that period granted without hesitation, and this passed with the rest, and was approved on the 2d of March, 1837. The application for a charter had been made in good faith, and with the full expectation of immediately organizing an institution under it; but the revulsion which took place in business in 1837, fell with blighting influence upon private and public enterprises alike, and some of those who, the year before, had the means and the disposition to aid and handsomely endow the institution, here found themselves without the means of supporting their own families.

The plan for the immediate organization of the institution was therefore dropped, and no action took place under the charter before the summer of 1843.

Early in the autumn of that year the Faculty of the college was organized by the appointment of four professors; professor Blaney held the chairs of Chemistry and Materia Medica, the late professor McLean, that of Theory and Practice of Medicine, Dr. M. L. Knapp, that of Obstetrics and Diseases of Women and Children, whilst that of Anatomy and Surgery were combined together and given myself. The fees for the entire course were, at that time, $60.

The session commenced on the 4th of December, 1843, and continued sixteen weeks. This was before the erection of any building, and the lectures were delivered in two small rooms, procured for the purpose, on Clark street. The number of students attendant upon this course, was twenty-two, and although there were several applicants for graduation, but a single degree was conferred. The first graduate of the institution, being William Butterfield, son of the late Hon. Justin Butterfield, of this city.

I have said that the undertaking was regarded by many at this time as premature, and it may be added that the preparations for this first course of lectures were made, in haste, and very imperfectly; and that the lectures of the institution were commenced one year sooner than had been intended in consequence of a prospectus for a course of lectures at La Porte, Indiana, and another for a course at St. Charles, in this State, having been issued; it being deemed not advisable to allow the schools organized at those points, to possess the advantage of priority of organization.

During the summer of 1844, the building which we used from that time to the close of the last course of lectures, was erected, the site being the same which we now occupy. The cost of this building which was was about thirty-five hundred dollars, was defrayed partly by loans and part by subscriptions, and the remainder was made up by the faculty themselves. The lot on which the building was situated was a donation from several owners of property on the North side of the river.

The second annual course of lectures commenced under circumstances far more auspicious than the first. The building which had been erected was found convenient and suitable to the wants of the class. The course on the Institutes and Practice of Medicine was given by Dr. Austin Flint, of Buffalo, that on Obstetrics and the Disease of Women and Children, by Dr. Graham M. Fitch, of Logansport, Ind., Dr. Blaney gave the course on Chemistry and Pharmacy, Dr. McLean, having been transferred to the Chair of Materia Medica and Therapeutics, Dr. Herrick gave the lectures on Anatomy, although not formllay elected to that chair until the following year. The course on Surgery was given as at the present time. The number of students in attendance was forty-six, upon eleven of whom, the degree of Doctor of Medicine was conferred, at the close of the term.

It is but justice to say in regard to this, which may truly be considered as the first full course of lectures in the Institution, that the character of the teachers, and the thoroughness of their instruction, were such as to command for the Institution, the respect of the public and the profession.

It is probable that no course given in the college, since its establishment, has given more entire satisfaction, or been really more advantageous to the students. It was during this course that the college clinique was first put in successful operation.

In the summer of 1845, Dr. Fitch was transferred to the chair of Institutes and Practice of Medicine,—rendered vacant by the retirement of Dr. Flint—and Dr. John Evans, elected to the chair of Obstetrics, and Diseases of Women and Children, which he has occupied to the present time. Dr. Herrick was elected to the chair of Anatomy, the course on which had been given by him the preceding winter. In other respects the faculty remained unchanged. The number of students in attendance was fifty, and the number of graduates ten.

For the course of 1846-47, the faculty remained unchanged; the number of students in attendance was seventy—the number of graduates sixteen. During this course the students had, for the first time, the advantages of clinical instruction in a hospital, established by the public authorities.

For the session of 1847-48, there was no change in the faculty. The number of students in attendance was one hundred and forty —the number of graduates thirty-three.

During the session of 1848-49, the course on Principles and Practice of Medicine, was given by Thomas Spencer: Dr. Fitch, having resigned the chair. A new chair of Physiology and Pathology was erected, to which Dr. N. S. Davis was elected. The number of students in attendance was one hundred—the number of graduates nineteen.

For the course of 1849-50, no change took place in the faculty, except the transferance of Dr. Davis to the chair of Institutes and Practice of Medicine; rendered vacant by the resignation of Dr. Spencer, and thus organized the faculty has continued its labors, until the commencement of the present year, when Dr. Herrick was, at his own request, transferred to the chair of Physiology and Pathology, Dr. J. W. Freer, elected to the chair of Anatomy, and Dr. H A. Johnson, to that of Materia Medica and Therap utics, rendered vacant by the resignation of Professor McLean.

The average number of students attending during the past five years, has been about one hundred and ten—of graduates about forty. The agregate number of students who have attended since the organization of the college, one thousand, one hundred and eight, and the alumni of the Institution number three hundred and eighteen, exclusive of honorary and ad-cundum degrees. *

---

* NOTE.

| | | | | | | |
|---|---|---|---|---|---|---|
| 1st | Course, | 1843-44, | Students | 22, | Graduates | 1. |
| 2d | do. | 1844-45, | do. | 46, | do. | 11. |
| 3d | do. | 1845-46, | do. | 50, | do. | 10. |
| 4th | do. | 1846-47, | do. | 70, | do. | 16. |
| 5th | do. | 1847-48, | do. | 140. | do. | 33. |
| 6th | do. | 1848-49, | do. | 100, | do. | 19. |
| 7th | do. | 1849-50, | do. | 104, | do. | 42. |

I have already said, that the preparations for the course of lectures for 1843 were made in great haste, and that the course was commenced sooner than was intended, on account of efforts which were made to establish schools in several neighboring villages. The late lamented Dr. Drake, who visited this city in the summer of 1844, expressed the opinion in a published letter, that Medical Schools ought to be located in the cities of Cincinnati, Louisville, St Louis, Chicago and Cleveland, in order to supply the wants of the West; and that the attempts to build up medical schools in small cities or in greater numbers than these, would be calculated to injure the profession. Coinciding fully in this view, it was thought advisable to organize the faculty of this Institution at the earliest practicable period.

Attempts were being made in the year 1843, to establish medical schools at St. Charles, and Jacksonville, in this State, and at La Porte, Indiana.

The effort at St. Charles entirely failed, and the students who had been assembled there, to the number of fifteen or twenty, dispersed; eight or ten of them came and attended the first course in this College. The school at Jacksonville was partially successful—several courses of lectures were given there, and in 1847 the class numbered nearly fifty students. The medical department was however closed soon after for want of sufficient encouragement.

The school at La Porte also attained a considerable degree of success. It was organized as early as 1842 or 1843, and on the failure of the effort to establish a school at St. Charles, the two Faculties were united at La Porte, and by the most untiring efforts, succeeded in drawing together a class of about a hundred

| | | | |
|---|---|---|---|
| 8th Course, | 1850-51, | Students 125, | Graduates 44. |
| 9th do. | 1851-52, | do. 105, | do. 30. |
| 10th do. | 1852 53, | do. 108, | do. 34. |
| 11th do. | 1853-54, | do. 122, | do. 37. |
| 12th do. | 1854-55, | do. 116, | do. 41. |
| | | 1118. | 318. |

students. This school, however, was also discontinued for want of support, in the year 1848.

Since then various efforts have been made to establish Medical Institutions at Indianapolis, Rock Island, Davenport, Keokuk and Evansville, which have either proved entire failures, or been, at best, but partially succssful; so that the prospect at the present time seems to be, for several years at least, that the Medical Schools of the North West will be restricted very nearly to the number indicated by Dr. Drake.

There is a subject which must not be omitted in the history of the College—not that it is intrinsically important but because it has been the subject of unjust animadversion upon the one hand, and of undue laudation upon the other. I allude to the reduction of Fees. This step was taken in the summer of 1849, and without entering into the details of the circumstances which led to the adoption of the measure at that particular time, it is sufficient to state here, that the fees of this College, after the reduction, were still higher than those of the Institution with which we were directly in competition, and that they are so at the present time. The Faculty of this College, while it has been progressive in what relates to science and its advancement, has been conservative in what relates to the honor and interest of the profession; yet while deploring the latitude which certain schools have permitted to themselves in many respects, we have not thought it incumbent on us to adopt a system of rigor in regard to fees, which would necessarily drive young men away from this, the natural seat and center of Medical Instruction in the North West, and thus build up schools in various country villages.

There may have been those among us, who allowed themselves for a moment to hope that placing Medical Schools under the patronage of the State, and making the institution free, while the courses were made more thorough, and the requirements for graduation much greater, would be the means of effecting that improvement in the profession, admitted on all hands to be so desirable. I confess myself to have been of the number who favored this view, but I have not the pretension to have learned nothing by experience, and to have always retained the same opinion. On the contrary the events of the last few years have been full of instruction for me, and the trial which has been made of this system in the state of Michigan, has convined me that, however imperfect medical schools may be, as commonly organized, those supported by the State, and controlled by public officers will not be less so, and that no improvement is to be expected from the State governments who are themselves unenlightened on the subject of medical instruction.

The result of our course in regard to fees, has been thus far extremely satisfactory. If our labors as teachers have not been liberally paid, neither have those of others, with few exceptions, who have attempted to build up medical schools, and while in other cities not more favorably situated than this, we have seen medical schools multiplying and some even established under the auspices of charlatans: in this region, they have diminished in numbers, and medical teaching has been more and more concentrated in this city.

I wish here particularly to call attention to the fact that the reduction of fees has not as was predicted been followed by an increase in the number of students. The largest class which has ever attended the lectures in this Institution was in the session of 1847 and '48, when there was a large and flourishing school in the neighborhood, and when the fees for the course were seventy dollars. This change is due mainly to the fact that the practice of drumming up students and enticing young men into medical colleges from the plough and mechanical pursuits, has in this vicinity been discontinued.

The building in which we are at present assembled, has been erected by the Faculty without aid from any source, and will cost when completed with the necessary fixtures about ten thousand dollars. The Faculty have not been desirous, even if their means had permitted it, of expending a large sum of money in the erection of a more elegant and expensive building for mere show, but have contented themselves with one sufficient for the present size of our classes, in which every arrangement for the health and comfort of the student has been properly attended to.

It will not I trust be expected of me, that I should speak of the comparative advantages which this college affords for attaining a good medical education—most of the professors are not unknown to you, and those who have recently been elected; although new to their present situations, are not by any means inexperienced as teachers, and I feel assured that I shall not be promising too much, when I say that we shall be able in all the several branches to afford you the means of sound and thorough instruction.

On the occasion of the dedication of our former building, I stated that it was the intention of those who had the direction of this Institution, to render it worthy of the city and the region of country in which it is placed. To do so, we are well aware that it must, in all essential particulars, be rendered fully equal to the best which exist in this country. From its first commencement until the present time, the faculty have never ceased to devote their best energies to raise it to a position of eminence, and to render it worthy of the respect and confidence of the profession, and of the community in which it is placed. We have not ceased to teach sound principles of Medical Science, whether or not they conflicted with the popular doctrines of the day, and I think it would not be claiming too much to assert that the public has been equally benefited, and the practice of the profession greatly improved by the dissemination of correct doctrines from that Institution.

The circumstances in which we have at times been placed, have been of a nature, well calculated to discourage and disheartin us. Medical schools are not in any part of the country in great favor with the multitude. No powerful societies with wealth and influence rally to their support. Governments, who acknewledged their obligation to establish schools and colleges for instruction in other branches, arc seldon found to extend aid, to medical institutions. Too often the laws throw obstacles in their way, yet notwithstanding all the difficulties we have proceeded from the beginning with a steady purpose of endowing this city with an institution which should be as permanent as her growth, and in all respects worthy of her greatness, and we are proud to say that in this undertaking we have been aided and cheered by the smiles and encouragement of those whose sympathy and good opinion we most highly value. Persons distinguished for their intelligence, their works of charity, their social virtues, and their wealth, have not ceased to give as the support of their good wishes, their advice, and in case of need of more substantial aid. But most of all have we been encouraged by this, that in spite of all the difficulties which surrounded us, numbers of young men about entering the profession have annually sought our halls for instruction. They have imbibed here a tone of science, an enthusiastic devotion to its pursuit, which in numerous instances have raised them to honorable positions and rendered them ornaments to their profession and to society.

I am well aware that it is unnecessary for me to say anything to excite your enthusiasm in the pursuit of the profession which you have chosen. You have no doubt entered upon it with a determination to pursue it seriously, ardently, and become thoroughly versed in its principles. Your presence here, and the manner in which many of you have already devoted yourselves to the preliminary course, are sufficient evidence of the zeal by which you are inspired. But lest some among you from a sense of imperfect instruction, insufficient means, or obscurity of position, might despair of attaining to a high degree of excellence, let me give you the assurance that no degree of eminence or success, is beyond the reach of him who shall perseveringly and ardently devote his energies to its attainment.

The experience of many years enables me to assure you that for all the time and attention which you may bestow upon your professional studies, you will be abundantly repaid, and that every excellence you may attain to, is certain of being fully appreciated, and acknowledged by the public, that the study of the profession if pursued in a right spirit is elevating in its tendency, its practice, exercises, and strengthens at once the faculties of the mind and the benevolent sentiments of the heart,—it is highly honorable and useful, many of you may have cause to regret your neglect of it, but not one will ever have reason to regret the hours which he has spent in acquiring a knowledge of it.

In conclusion Gentlemen, you will allow me to express the gratification which I feel in common with my colleagues and the friends of the Institution, in being able to welcome you to these Halls. It is now fifteen years since I commenced Medical Instruction in the city of Chicago. The first course of private lectures which I gave upon Anatomy, was given to a class of six students in the back room of my office; the second course was attended by eleven students, and a second bench had to be added to the one used the previous winter to accommodate the class. That embryo amphitheatre has developed itself by successive steps, to the dimensions which you see before you. The Institution which was planted with so much care, and built up with so much labor, has now attained to the vigor of maturity, and sustains, and enlarges itself by its own inherent strength. In its success our best hopes have been realized, and our fears dispelled, and encouraged by the past we look forward to a career full of prosperity and usefulness. It may, indeed, meet with obstacles and varying degrees of fortune, for it has with truth been said that an Institution which has been planted, is like a ship entrusted to the sea, she may go forth under smiling skies, but in her course she must meet with storms, but although the waves may beat upon her, although the winds may rage around her, dark clouds gather above, or rocks obstruct her path, yet conducted by skilful pilots, she will pass unharmed through every danger, and arrive in safety at her destined port. The Medical College of Chicago, is chained to the destiny of a great and noble city, and so long as Chicago shall be the seat of commerce and intelligence, so long will our school be honored and sustained.

